# From the leaf to the gut and back again: using synthetic bacterial communities to trace the fate and influence of phyllosphere bacteria in an Arabidopsis–*Pieris brassicae* system

**DOI:** 10.1186/s40793-026-00947-y

**Published:** 2026-08-06

**Authors:** Moritz Müller, Maryse A. P. Huve, Michael Kunzler, Mitja N. P. Remus-Emsermann, Rudolf O. Schlechter, Luis R. Paniagua Voirol

**Affiliations:** Institute of Microbiology and Dahlem Centre of Plant Sciences, Department of Biology, Chemistry, Pharmacy, Freie Universität Berlin, Berlin, Germany

**Keywords:** Gut microbiota, Jasmonic acid, Lepidoptera, Phyllosphere microbiota, Synthetic community, Insect performance, Community diversity

## Abstract

**Background:**

The leaf surface, or phyllosphere, hosts abundant and diverse bacterial communities that interact with both the host plant and herbivorous insects, yet their collective influence on plant–insect interactions remains poorly investigated. We established a functionally gnotobiotic insect–plant system combining axenically grown *Arabidopsis thaliana*,* Pieris brassicae* larvae, and defined synthetic phyllosphere communities (SynComs) of increasing richness: SynCom5, SynCom10 and SynCom20, containing 5, 10 and 20 bacterial strains, respectively. We investigated how phyllosphere bacteria influence herbivore performance, plant defence responses, and bacterial colonisation of both leaves and the insect gut.

**Results:**

While larval weight tended to decrease with increasing bacterial community richness, only the most diverse SynCom (20 members) caused significant weight reductions without affecting survival. Plants harbouring the most diverse community showed enhanced jasmonic acid (JA) levels during feeding, whereas salicylic acid (SA) remained unchanged, suggesting the induction of herbivore-associated defence responses. Compared to plants experiencing no herbivory, feeding strongly reshaped bacterial colonisation on leaves, increasing total bacterial loads about fourfold and driving dominance of *Pantoea eucalypti* 299R as shown by 16S rRNA gene amplicon sequencing. Larvae acquired a distinct subset of bacteria, primarily recruited from the genera *Methylobacterium*, *Microbacterium*, *Williamsia*, and *Curtobacterium*.

**Conclusions:**

Phyllosphere bacteria can influence plant–insect interactions, with bacterial community richness associated with reduced *P. brassicae* larval performance and SynCom20 linked to stronger JA accumulation in leaves during feeding. Conversely, herbivory increased leaf bacterial loads, shifted leaf community composition, and filtered leaf-associated bacteria during passage into the larval gut. These findings establish this functionally gnotobiotic insect–plant system as a tractable model for dissecting reciprocal effects between resident phyllosphere bacteria and herbivory.

**Supplementary Information:**

The online version contains supplementary material available at 10.1186/s40793-026-00947-y.

## Introduction

The phyllosphere—the aerial surface of plants—represents one of the largest terrestrial habitats for microbial life on Earth, with leaves alone covering an estimated 6.4 × 10^8^ to 1 × 10^9^ km^2^ globally and hosting up to 10^7^ bacterial cells per gram of leaf tissue [1]. Leaves are consumed by a wide range of herbivorous insects, many of which are major pests in agricultural and natural ecosystems [2, 3]. The phyllosphere therefore represents an ecologically important interface where microbes and insects interact, with direct consequences for plant health and productivity.

Microbes and herbivorous insects can influence each other indirectly through the plant’s immune system, which integrates multiple inducible defence pathways that are largely regulated by phytohormones [4, 5]. Jasmonic acid (JA) typically mediates responses to chewing herbivores and necrotrophic pathogens, while salicylic acid (SA) mediates responses to biotrophic pathogens and piercing–sucking insects [4, 6, 7]. Because these pathways often act antagonistically, activation of one often suppresses the other, creating scope for plant-mediated microbe–insect interactions. Accordingly, microbes and herbivores influence each other by modulating JA–SA cross-talk [8]. For instance, when leaves are damaged by chewing herbivores, JA-mediated signalling suppresses SA-dependent defences, allowing biotrophic pathogens to proliferate [7, 9]. Some pathogens enhance herbivore performance by activating SA-mediated defences or, conversely, suppress feeding by producing JA mimics [9–11]. Insect mutualists, which can be either pathogenic or nonpathogenic to plants, may suppress plant anti-herbivore defences by eliciting SA responses or by preventing recognition of the insect, thereby reshaping insect–plant interactions [12, 13]. Beyond pathogens and mutualists, nonpathogenic phyllosphere bacteria also elicit plant immune responses when they reach high densities [14–16]. Despite these insights, the broader ecological role of nonpathogenic phyllosphere bacteria in shaping plant–insect interactions is still poorly understood.

Besides hormonal cross-talk, herbivorous insects and phyllosphere bacteria also interact through a range of direct physical and ecological processes. Herbivory damage releases nutrients into the otherwise nutrient-poor phyllosphere, creating transient hotspots that promote bacterial growth and entry into the apoplast [17, 18]. Insects further impact microbial dispersal by vectoring bacteria onto leaves via frass, saliva, or body surfaces [12]. These effects operate alongside other ecological factors shaping the leaf microbiota, including host identity, plant immune activity, cuticle composition, and plant metabolism [19]. Together, these processes shape the composition and dynamics of phyllosphere bacterial communities and may influence how microbes and herbivores affect each other through the plant.

Most studies on plant–microbe–insect interactions in the phyllosphere have focused on individual mutualistic or pathogenic species [12, 20, 21]. By contrast, the ecological roles of resident nonpathogenic microbial communities and their effect on plants and herbivorous insects remain poorly understood. This raises key questions: Do the composition and diversity of microbial communities influence herbivory, and how does herbivore feeding, in turn, restructure these communities? Do phyllosphere microbes affect insect performance indirectly by modulating host defences, or directly through ingestion and gut colonisation?

To address these questions, we established a functionally gnotobiotic insect–plant system combining *Arabidopsis thaliana* (thale cress), *Pieris brassicae* (Large White; Lepidoptera) larvae, and synthetic phyllosphere bacterial communities (SynComs) of different richness. *Pieris brassicae* is a specialist herbivore of Brassicaceae, widely distributed in Europe, Asia, and North Africa, and a major pest of crops such as cabbage, broccoli, and mustard [22]. Its larvae are foliar feeders that cause extensive tissue damage. Like most studied Lepidoptera, the larval gut of *P. brassicae* lacks a stable resident microbiota, with bacteria acquired transiently mainly through feeding [23, 24]. Under laboratory rearing conditions, the gut of *P. brassicae* is virtually sterile, with only minimal bacterial presence [24], making this species an ideal system for manipulating bacterial exposure. This functionally gnotobiotic insect–plant system enables investigation of how phyllosphere bacterial communities influence larval performance, plant responses, and microbial colonisation patterns within a defined tripartite plant–microbe–insect interaction.

To explore how resident phyllosphere bacteria shape the interplay between plants and herbivores, we combined insect performance assays, phytohormone analysis, and 16S rRNA gene amplicon sequencing. We investigated (1) how phyllosphere bacteria affect herbivorous insect performance, (2) how these bacteria modulate levels of anti-herbivore defence-associated phytohormones, (3) how herbivory shapes the leaf-associated bacterial community, and (4) which of these bacteria are recruited by the larval gut environment. This work uncovers emergent properties of plant–microbe–insect interactions in the phyllosphere and establishes a tractable functionally gnotobiotic model for dissecting their underlying mechanisms.

## Materials and methods

### Plant growth conditions

*Arabidopsis thaliana* Col-0 seeds were surface-sterilised with 70% v/v ethanol for 2 min, followed by a 50% v/v household bleach solution (DanKlorix, Germany) containing 0.2% v/v Tween-20 for 7 min. Seeds were thoroughly washed with sterile water three times and stratified for two days at 4 °C. Single seeds were sown onto approximately 0.2 cm thick, 0.5 cm × 0.5 cm wide slices of half-strength Murashige and Skoog (1/2 MS) medium (Duchefa) with 1% w/v plant agar (Duchefa) or onto cut-off pipette tips filled with the same medium [16], transferred into Petri dishes wrapped with Parafilm (Bemis Parafilm PM-996) and put into a plant growth chamber (poly klima GmbH, M5Z-TDL+) at 21 °C, 80% relative humidity, ~120 µmol m^−2^ s^−1^ light intensity, and a 11 h light/13 h dark photoperiod. Then, nine-day-old seedlings were transferred into sterile plant tissue culture boxes (size: 7 × 7 × 8 cm) (Bioworld Magenta GA-7 Plant Culture Box) filled with 90 g zeolite (0.2–0.5 mm grain ZeoLith Klinoptolith, labradorit.de) supplemented with 40 ml of sterile 3/4 MS medium (Duchefa). Box lids had four holes with a diameter of roughly 0.5 cm sealed with a double-layered micropore strip (3M Deutschland GmbH) to prevent contamination while allowing for air exchange. Four seedlings were transferred under aseptic conditions into each box and put back into the growth chamber. For the SynCom experiments, each box, containing four plants and later three larvae, constituted one biological replicate.

### Synthetic bacterial communities

SynCom composition was selected from a pool of 20 bacterial strains from prevalent genera of the *A. thaliana* phyllosphere microbiota (Additional file 1: Table S1). All strains used were originally isolated from the phyllosphere, are nonpathogenic to *A. thaliana*, and represent major phylogenetic and functional groups of the leaf microbiota [25–27]. To initially test the effect of bacterial community richness on larval performance, we designed SynComs from the species pool with three richness levels: SynCom5, SynCom10, and SynCom20, containing 5, 10, and 20 strains, respectively. For SynCom5 and SynCom10, members were randomly drawn out of the total pool and therefore unique in their composition in each independent experiment (Additional file 1: Fig. S1), while SynCom20 remained constant as a community with the maximal functional potential and redundancy from this pool.

Strains were individually grown in Reasoner’s 2A (R2A) medium or on R2A agar (HiMedia) for 1–4 days at 30 °C. Cultures were harvested by centrifugation for 7 min at 8,500 × *g*, resuspended in 10 mM MgCl_2_, diluted to a predetermined optical density at 600 nm (OD_600_), and assembled in equal CFU ratios (Additional file 1: Table S1). SynComs were stored as glycerol stocks (20% v/v glycerol) at –80 °C in either 0.5- or 1-ml aliquots.

### Inoculation of *A. thaliana* with bacterial communities

**Fig. 1:**
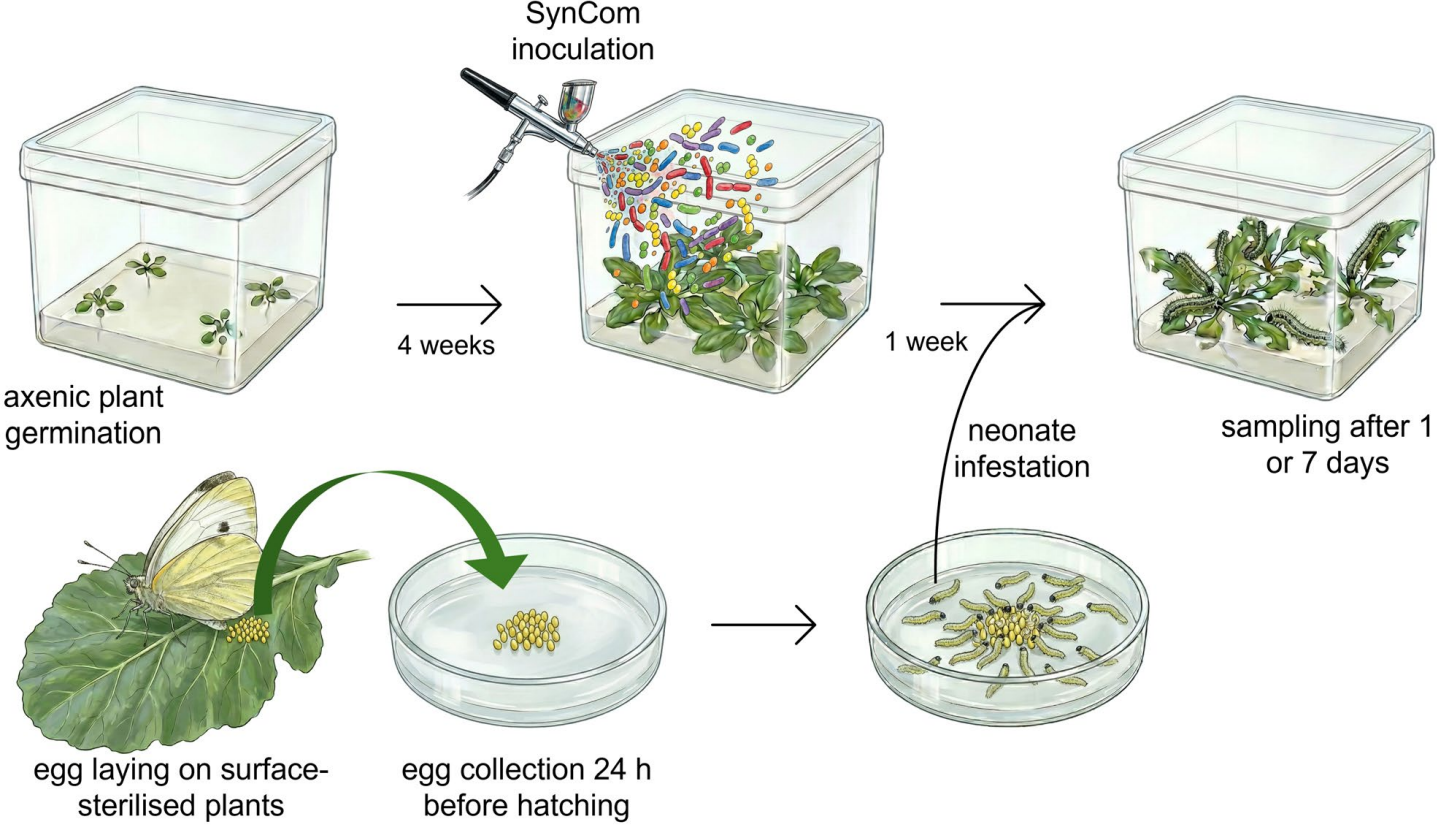
Functionally gnotobiotic plant–insect system. *Arabidopsis thaliana* plants were grown under axenic conditions on a zeolite–MS substrate for four weeks in sealed plant tissue culture boxes. Plants were inoculated with SynComs by foliar spraying. Control plants were mock-treated with PBS. In parallel, *Pieris brassicae* eggs were collected from surface-sterilised Brussels sprout plants, removed from the leaves one day before hatching, and placed in sterile Petri dishes to obtain neonates for the experiments. Eight days after plant inoculation, *P. brassicae* neonates were introduced into the system under aseptic conditions. As defined in the Methods, the system is considered functionally gnotobiotic because axenically grown plants were exposed to defined bacterial communities or mock treatment, while any larva-associated background bacteria were expected to remain minimal. After the feeding period (one or seven days depending on the experiment), leaves and larvae were sampled for bacterial quantification, community profiling, and phytohormone quantification. One box containing four plants and three larvae constituted one biological replicate

To ensure inoculum consistency and minimise variability, all samples receiving a given SynCom composition were inoculated using frozen SynCom aliquots with a predetermined OD_600_ derived from the same batch. The direct use of frozen aliquots for plant inoculation has been demonstrated as an effective approach [28]. Glycerol stocks were thawed and centrifuged at 8,500 × *g* for 7 min. The supernatant (containing the residual glycerol and MgCl_2_) was discarded, and the pellet was resuspended in 1 ml phosphate-buffered saline (PBS; 8 g/L NaCl, 1.44 g/L Na_2_HPO_4_, 0.24 g/L KH_2_PO_4_, 0.2 g/L KCl; pH 7.4). Bacterial suspensions were then diluted to an OD_600_ of 0.005. Plants were sprayed four weeks after sowing with an airbrush (Harder & Steenbeck Ultra) at 10 psi using a sterile plant culture box lid containing a single micropore-sealed hole (Fig. 1). Before and between treatments, the airbrush was sterilised with 70% v/v ethanol and rinsed with sterile PBS. All boxes received 200 µl of either the SynCom suspension or sterile PBS alone (mock control), ensuring that the carrier solution was identical across treatments.

Throughout the manuscript, we use the term gnotobiotic in a functional sense. Axenically grown plants were either mock-treated or inoculated with defined SynComs, allowing plant-associated microbial exposure to be experimentally controlled. We acknowledge that the introduction of neonate larvae may occasionally introduce low levels of environmental bacteria into the system; this background is expected to be minimal, as *P. brassicae* larvae harbour only negligible bacterial loads under our laboratory rearing conditions [24]. Thus, “gnotobiotic” here refers to a functionally gnotobiotic plant–insect system that enables comparison between low-background mock-treated controls and plants colonised by defined phyllosphere SynComs.

### Insect rearing and neonate collection for experiments

*Pieris brassicae* larvae were reared on greenhouse-grown Brussels sprout plants (*Brassica oleracea* var. *gemmifera*) in a temperature-controlled room at 22 °C, 75% relative humidity, ~120 µmol m^−2^ s^−1^ light intensity, and an 18 h light/6 h dark photoperiod, in 50 × 50 × 60 cm rearing cages containing approximately 100 individuals, until pupation and emergence. Adults were transferred to a greenhouse (20–24 °C day / 18–22 °C night, 75% relative humidity, ~ 120 µmol m^−2^ s^−1^ light intensity, 16 h light/8 h dark photoperiod) and fed with a 15% v/v aqueous honey solution. For egg collection, seven-week-old Brussels sprout plants were prepared by spraying the underside of the leaves with 70% v/v ethanol and allowing them to dry. One plant was then placed in a cage containing one-week-old butterflies and left overnight for oviposition. The egg-laden plant was then transferred to the larval temperature-controlled room. To prevent the hatching larvae from feeding on the egg-ladenplants, eggs were gently removed from the leaves using sterilised tweezers and placed in sterile Petri dishes one day before hatching (four days after egg laying). On the day of hatching, timed to coincide with eight days after SynCom inoculation, all neonates were pooled and randomly assigned to the plant treatments. Neonates were carefully transferred using sterilised paintbrushes, with three neonates placed per plant tissue culture box (Fig. 1). This handling procedure was designed to minimise microbial carry-over, and previous work showed that laboratory-reared *P. brassicae* larvae harbour only negligible bacterial loads under controlled conditions [24]. Each box constituted one biological replicate.

### Assessment of larval growth in the functionally gnotobiotic insect–plant system

To assess larval growth in the functionally gnotobiotic insect–plant system, sterile *A. thaliana* Col-0 seeds, prepared as described above, were sown on agar. After nine days, part of the seedlings was transplanted into pots (size: 9 × 9 × 8 cm) containing a 3:1 mixture of steamed soil (Einheitserde Typ P soil, Kausek) and vermiculite (Kausek). The remaining seedlings were transferred into sterile plant tissue culture boxes as described above. As in the plant tissue culture boxes, four plants were transferred in each pot. Plants were used 36 days after sowing, and four neonate larvae were placed per pot. Larval biomass and survival were evaluated after seven days of feeding on plants grown in parallel either in pots or in tissue culture boxes. To that end, one neonate was placed on each plant within the boxes or pots, for a total of four neonates per box or pot. To prevent larvae from escaping, pots were enclosed in microperforated bags (Packseller) and sealed with tape. Plants and boxes were then placed in a plant growth chamber (poly klima GmbH, M5Z-TDL+) set to 21 °C, 80% relative humidity, ~120 µmol m^−2^ s^−1^ light intensity, and an 11 h light/13 h dark photoperiod. Each box or each pot constituted one biological replicate. All larvae from each replicate were weighed together using a precision balance (Precisa H_225SM-DR; resolution 0.01/0.1 mg) and the total weight was divided by the number of weighed larvae. The number of larvae per replicate was also recorded.

### Larval sampling

For all SynCom experiments, *P. brassicae* larvae were sampled one day after being placed on the plants for phytohormone measurements, or after seven days for all other experiments (Fig. 1). Larvae from each box were pooled and treated as one biological replicate. The number of surviving larvae and their total weight were recorded for each box using a precision balance (Precisa H_225SM-DR; resolution 0.01/0.1 mg). Larvae were placed at −20 °C for 2 min to induce lethargy, then homogenised in 250 µl PBS in 1.5 ml Eppendorf tubes using sterilised metal pestles. An aliquot of the homogenate was stored at −20 °C for DNA extraction. From 40 µl of homogenate, a tenfold serial dilution was prepared and plated on R2A agar using a 96-pin replicator to quantify total CFU per larval weight.

### Leaf sampling

Leaves were collected seven days after larval placement, in parallel with larval sampling. Two larvae-fed leaves per plant were taken from each box, excised at the petiole. Leaves from the same box were pooled in sterile 1.5 ml Eppendorf tubes and treated as one biological replicate. Fresh weight was recorded using a precision balance (Precisa H_225SM-DR; resolution 0.01/0.1 mg). To detach phyllosphere bacteria, each tube was filled with 1 ml PBS, sonicated for 5 min (75% ultrasound efficiency; EMAG Emmi-12HC), and vortexed for 10 s. A tenfold serial dilution of each leaf wash was then prepared and inoculated onto R2A agar using a 96-pin replicator. Each sample was plated in duplicate and incubated at 30 °C. Total CFU counts were normalised to leaf fresh weight, and the remaining leaf wash was stored at −80 °C for DNA extraction.

### DNA extraction and 16S rRNA gene amplicon sequencing

Leaf, larval, and SynCom20 inoculum samples were collected from a herbivory experiment in which axenically grown *A. thaliana* plants were inoculated with SynCom20; eight days later, *P. brassicae* neonates were introduced and allowed to feed for seven days before sampling. DNA was extracted using the MasterPure™ Complete DNA & RNA Purification Kit, with adjusted volumes of Tissue and Cell Lysis Solution. For larvae, 300 µl lysis solution was added to 150 µl homogenate and transferred to a 2-ml tube containing seven sterile zirconia beads. For leaf samples, 450 µl lysis solution was added per sample. For the SynCom20 inoculum, glycerol stocks were centrifuged at 8,500 × *g* for 7 min in a 2-ml tube; the supernatant was discarded, and seven zirconia beads plus 450 µl lysis solution were added. Larval and inoculum samples were homogenised in a Retsch MM400 bead mill for four cycles of 2 min at 30 Hz. Extraction negative controls (kitome) consisting of reagent-only tubes were processed in parallel to the leaf and larval samples. Subsequent steps followed the kit protocol. DNA quality and quantity were measured using a DeNovix DS-11 FX+ spectrophotometer/fluorometer with the dsDNA Broad Range kit. Samples were stored at −20 °C until processing.

Amplicon sequencing of the V3–V4 region of the 16S rRNA gene was done using the primers 341F (5′-CCTACGGGNGGCWGCAG-3′) and 806R (5′-GGACTACNVGGGTWTCTAAT-3′) with barcodes for pooling [29]. PCR reaction mix was prepared per sample with 10 µl 5× Phusion GC Buffer, 1 µl 10 mM dNTPs, 2.5 µl forward and reverse primers at 10 µM, 50 ng template DNA, 1.5 µl DMSO, 0.5 µl Thermo Scientific Phusion High-Fidelity DNA Polymerase, and filled up to 50 µl with water. The PCR cycling protocol included an initial denaturation for 30 s at 98 °C, then 30 cycles of denaturation at 98 °C for 10 s, annealing at 58 °C for 30 s, and extension at 72 °C for 30 s. A final extension was done at 72 °C for 5 min. Each PCR run included a no-template control (NTC) and a positive control containing bacterial DNA from an aliquot of the stocked inoculum.

Amplicons were verified by agarose gel electrophoresis. PCR run quality was assessed with kitome, positive, and NTCs processed in parallel with biological samples. Samples were purified using the Monarch PCR & DNA Cleanup Kit (New England Biolabs). DNA quality and concentration of purified amplicons were assessed on a DeNovix DS-11 FX+ spectrophotometer/fluorometer, and samples were pooled and concentrated to meet sequencing requirements. The pooled amplicon library was then sent to Novogene (Germany) for library preparation (PCR-free protocol) and sequencing on an Illumina NovaSeq X Plus platform (250-bp paired-end reads).

### Analysis of 16S rRNA gene amplicon data

Paired-end reads were merged using PEAR with a minimum overlap of 10 bp and a *p*-value threshold of 0.01 [30]. Merged reads were demultiplexed using Cutadapt, and sequences for each sample were quality-filtered using USEARCH v11.0.667 [31], retaining reads >350 bp with Phred scores >19. Filtered reads were dereplicated and clustered into OTUs at 98% sequence similarity. Representative sequences and OTU abundance tables were generated for taxonomic classification and diversity analysis, respectively. This analysis was performed with the High-Performance Computer “CURTA” of FUB-IT, Freie Universität Berlin [32].

Further analyses were conducted in RStudio [33], using the DECIPHER, Phyloseq, and Biostrings packages [34–40]. OTUs were classified using the SILVA rRNA database (version 138.2, NR99 release), to identify and remove chloroplast, mitochondria, and unclassified OTUs (Additional file 1: Fig. S2). Given that members of SynCom20 have been whole-genome sequenced, we further identified them by global alignment of OTU sequences with a custom V3-V4 reference database, constructed from the V3–V4 sequences of each of the 16S sequences, using USEARCH v11.0.667 [31], with at least 98% sequence identity and full-length coverage. For each query, only the best hit was reported. Pairwise sequence identity among all SynCom20 V3–V4 sequences was calculated to evaluate the discriminatory power of this region at the strain level, with strains sharing >98% of sequence identity in this region being potentially unresolvable at this taxonomic resolution (Additional file 1: Fig. S3).

Phylogenetic relationships among SynCom20 members were generated based on orthogroup inference of annotated whole-genome sequences using OrthoFinder [41, 42]. Phylogenetic structure was evaluated by calculating the Faith’s phylogenetic diversity (PD) and mean pairwise phylogenetic distances (MPD), and visualised using the packages ggtree, picante, ape, and ggstance [43–50].

Analysis of the bacterial communities was done with the R package vegan [51]. To minimise sampling bias, samples with fewer than 250 reads, including the negative controls, were excluded from further analysis, resulting in ≥99.5% Good’s coverage across all retained samples. OTU tables were rarefied 100 times to 250 reads per sample, mean abundances were determined, and OTUs with mean of zero were removed. Alpha diversity was estimated by richness, evenness, and Shannon diversity index.

Beta diversity was estimated on Bray–Curtis distances and used to visualise differences in community composition among treatments using non-metric multidimensional scaling (NMDS). To identify taxa whose abundance was associated with a treatment, OTU vectors were fitted onto the NMDS ordination space (*envfit*, vegan package), retaining only significant correlations at *p* <0.05. In addition, differential abundance analysis on relative abundances of SynCom20 was performed using DESeq2 to identify taxa that are significantly enriched or depleted across treatments [52]. *P*-values of fold changes were corrected using Benjamini–Hochberg (BH) procedure to control for false discovery rates.

### Phytohormone extraction and analysis

Leaf material for phytohormone quantification was collected eight days after bacterial inoculation and before placing the larvae (T0), and 24 h later (T1). One leaf was sampled per plant from each replicate box, prioritising larvae-fed leaves. Plant material was sampled in 2 ml tubes containing four zirconia beads, and immediately snap-frozen in liquid nitrogen before storing at −80 °C until processing. Phytohormones were extracted using a protocol outlined by Wang et al. 2007 [53]. Briefly, frozen samples were weighed and homogenised in 1 ml of ethyl acetate and 2 µl of internal standards containing deuterated phytohormones (10 ng/µl D4–salicylic acid [SA], 10 ng/µl D6–abscisic acid [ABA], 30.2 ng/µl D6–jasmonic acid [JA], 10 ng/µl D6–jasmonoyl-isoleucine [JA-Ile]; HPC Standards GmbH). Homogenisation was done in cooled adapters on a Retsch MM400 bead mill for nine cycles of 20 s each at 30 Hz, followed by centrifugation at 13,000 × *g* for 10 min at 4 °C. Supernatants were transferred to new tubes, while 1 ml ethyl acetate was added to the pellets and the extraction step was performed a second time without internal standard. The supernatants from the second extraction round were pooled with the first ones and concentrated under vacuum (Savant DNA Speed Vac DNA 110). The concentrates were re-eluted in 300 µl of re-elution buffer (70% methanol, 0.1% formic acid), vortexed for 10 min at 22 °C and 1,500 rpm on the BIOER Mixing Block MB-102, and centrifuged for 20 min at 13,000 × *g* at 22 °C, before 200 µl supernatant was transferred into an HPLC vial. Extraction blanks were processed alongside samples. Quantification of JA, JA-Ile, ABA, and SA was performed using liquid chromatography–electrospray ionisation–ion mobility spectrometry–tandem mass spectrometry (LC-ESI-IMS/MS; Waters UPLC-Synapt G2-S HDMS).

Peak identification and integration were done in MassLynx (Waters). Phytohormone concentrations were normalised to their corresponding deuterated internal standards and the leaf fresh weight. JA-Ile was normalised by the ratio of the deuterated JA-Ile and the deuterated JA-Ile impurity. Finally, phytohormone levels at T1 were normalised to the corresponding T0 value from the same biological replicate. Data from two independent experiments were analysed jointly.

### Statistical analysis

All statistical analyses, regression models, and data visualisation were performed in RStudio [33]. For the SynCom experiments, the plant tissue culture box was used as the unit of replication. In the comparison between pot and tissue culture systems, each pot or box constituted one biological replicate. Plots were generated using the packages ggplot2, ggsignif, smplot2, and patchwork [54–57]. Data wrangling was carried out using the tidyverse [58].

Unless stated otherwise, (generalised) linear mixed-effects models ((G)LMM) were fitted to account for variation among independent experiment replicates, using the nlme or glmmTMB package [59–61]. Depending on the experiment, response variables included larval weight, log_10_-transformed CFU counts, larval survival, or normalised phytohormone levels. SynCom treatments and/or larval feeding were treated as fixed effects, while independent experiments were included as a random intercept. Model assumptions were evaluated by visual inspection of residual plots. Model fit was assessed by inspecting simulation-based scaled residuals using the DHARMa package. Outliers were identified using the outliers and testOutliers functions on the simulated residuals of each model. Observations identified as outliers were excluded only from the model in which they were detected, resulting in the removal of one observation from the phytohormones JA and two from ABA. The effect of different treatments was evaluated using analysis of variance (ANOVA) for linear mixed models or a Type II Wald chi-square test for generalised models. Then, estimated marginal means were calculated using the emmeans package [62], and pairwise comparisons were adjusted with the BH procedure for multiple comparisons. Compact letter displays were generated to indicate statistically different treatment groups.

For comparisons between two groups, parametric or non-parametric tests were applied after inspecting assumptions of normality (Shapiro–Wilk normality test) and homoskedasticity (Levene’s test) using the rstatix package [63]. Differences in larval biomass between pot and tissue culture box systems were evaluated using a Wilcoxon signed-rank test, whereas larval survival across these growth systems was compared using Fisher’s exact test. Bacterial abundance on the leaves after a seven-day *P. brassicae* feeding period on either SynCom20-inoculated or mock-treated axenically grown control plants was compared using Student’s *t*-test.

To determine whether bacterial community composition differed among sample types (inoculum, fed leaf, non-fed leaf, larvae) after the feeding period, PERMANOVA was performed using the *adonis2* function in the vegan package on a Bray-Curtis distance matrix [64]. Differences in alpha diversity metrics (richness, evenness, Shannon diversity) and mean pairwise phylogenetic distances (MPD) were analysed using ANOVA followed by Tukey’s *post hoc* test.

## Results

### Bacterial abundance on leaves and larvae across communities of different richness and its effect on larval performance

We established a functionally gnotobiotic insect–plant system to study the interactions between *A. thaliana*, phyllosphere bacteria, and *P. brassicae*. To confirm that the system was suitable for larval rearing, we compared *P. brassicae* performance on gnotobiotic plants with that on plants cultivated in pots. While larvae were on average larger in pots compared to the functionally gnotobiotic system, larval survival did not differ between conditions (Additional file 1: Fig. S4).

To assess the effect of community richness on the interaction between *A. thaliana* and *P. brassicae*, plants were inoculated with SynComs of different richness. A pool of 20 phyllosphere strains was selected to capture the taxonomic diversity of natural leaf communities (Additional file 1: Table S1). Five or ten strains were randomly selected from the bacterial pool to assemble SynCom5 and SynCom10 for each independent experiment, respectively, to focus our investigation on richness rather than effects of individual strains (Additional file 1: Fig. S1). A SynCom composed of the total strain pool was included to capture the highest richness level (SynCom20). Eight days after spray inoculation of plant leaves, *P. brassicae* neonates were introduced onto SynCom-inoculated or mock-treated axenically grown control plants, and after seven days of feeding, bacterial abundance on fed leaves and larvae, as well as larval performance, were assessed.

**Fig. 2:**
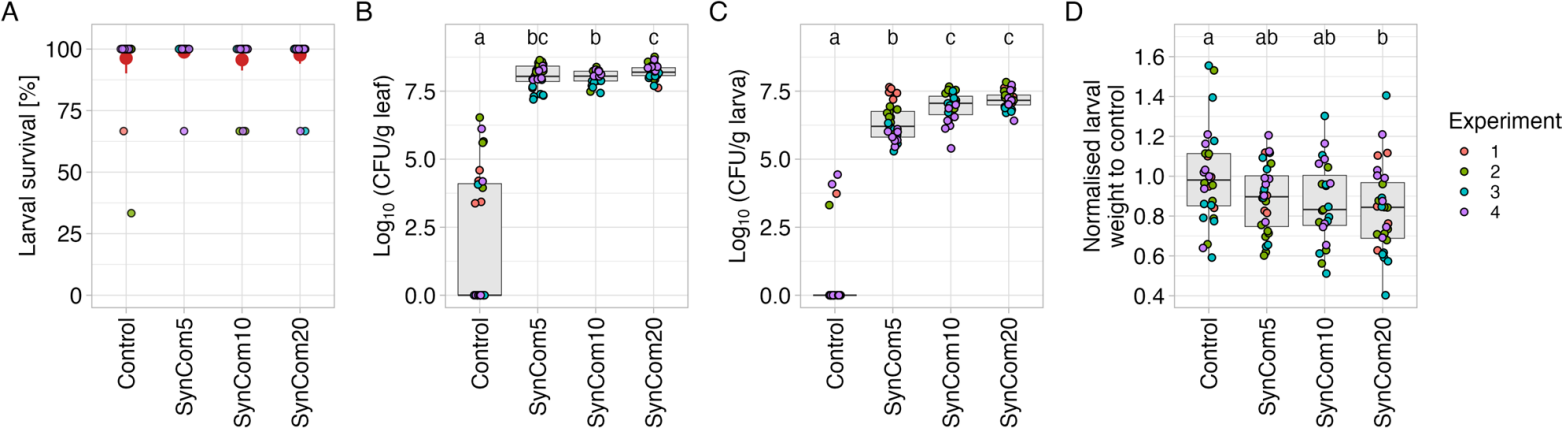
Effect of SynComs on bacterial colonisation and larval performance. Plants were inoculated with SynComs of increasing richness and incubated for eight days before neonates were placed to feed. After seven days of feeding, bacterial abundance (CFU/g) and larval weight were measured. **A** Survival of larvae on mock-treated control plants or plants inoculated with SynCom5, SynCom10, or SynCom20. Mean and 95% CI from non-parametric bootstrap resampling are shown in red. **B** Bacterial abundance on leaves and **C** in *Pieris brassicae* larvae fed on mock-treated control plants or SynCom-inoculated plants. For each panel the number of replicates is the same: control (*n* = 28), plants inoculated with SynCom5 (*n* = 28), SynCom10 (*n* = 23), or SynCom20 (*n* = 28). One biological replicate corresponds to all sampled leaves or all sampled larvae from a single box. SynCom10 was not included in Experiment 1. Low bacterial counts in mock-treated controls likely reflect occasional introduction of environmental bacteria after neonate transfer; these levels were substantially lower than those observed in SynCom-inoculated treatments. **D** Normalised larval weight relative to the mean weight of the control samples within each experiment. Different letters indicate significant group differences among treatments (*p*_adj_ < 0.05) based on linear mixed-effects models followed by Benjamini–Hochberg-adjusted pairwise comparison of estimated marginal means

After seven days of feeding in the functionally gnotobiotic system, larval survival rate was similar across treatments. On mock-treated control plants, an average of 96% of larvae survived, while mean survival on plants inoculated with SynCom5, SynCom10, and SynCom20 was 99%, 96%, and 98%, respectively, with no significant differences among treatments (Fig. 2A, χ^2^ = 1.54, df = 3, *p* = 0.67).

Phyllosphere bacterial abundance on *A. thaliana* leaves was similar across SynCom treatments, with only a modest increase in SynCom20 compared to SynCom10 (Fig. 2B, *t*(83) = 2.2, *p*_adj_ = 0.045). Median bacterial loads were 1.6 × 10^8^, 1.2 × 10^8^, and 1.9 × 10^8^ CFU per g of leaf fresh weight for SynCom5, SynCom10, and SynCom20, respectively. The effect of SynCom richness on bacterial loads was significant (ANOVA; *F*_3,83_ = 209.5, *p* < 0.001). All plants that were inoculated with SynComs carried significantly higher bacterial loads than mock-treated controls (*p*_adj_ < 0.001). The low bacterial counts detected in mock-treated controls indicate that larval introduction occasionally introduced background bacteria. However, these levels were substantially lower than those recovered from SynCom-inoculated treatments, preserving a clear contrast between mock-treated controls with minimal background bacterial presence and plants colonised by defined bacterial communities.

Bacterial abundance in *P. brassicae* larvae differed significantly among SynCom treatments (ANOVA; *F*_3,100_ = 409.1, *p* < 0.001), with median loads of 1.6 × 10⁶, 1.1 × 10⁷, and 1.6 × 10⁷ CFU per g of larval fresh weight after feeding on plants inoculated with SynCom5, SynCom10, and SynCom20, respectively  (Fig. 2C). All SynCom treatments carried significantly higher bacterial loads than the mock-treated control (*p*_adj_ < 0.001). SynCom10 and SynCom20 had significantly higher bacterial loads than SynCom5 (*p*_adj_ < 0.01), whereas no significant difference was detected between SynCom10 and SynCom20.

Larval weight was significantly influenced by SynCom treatment (Fig. 2D, ANOVA on LMM; *F*_3,100_ = 3.62, *p* = 0.0157). Larvae fed on SynCom20-inoculated plants had a significantly lower weight than larvae fed on mock-treated control plants (Fig. 2D, *p*_adj_ = 0.0153). On average, larval weight decreased by 13.1%, 14.4%, and 17.6% when plants were inoculated with SynCom5, SynCom10, and SynCom20, respectively, compared to the mean larval weight on mock-treated control plants. However, no significant differences were detected among the three SynCom treatments (ANOVA on LMM, all comparisons: *p*_adj_ > 0.05). Larval weight correlated negatively with SynCom richness (*r* = −0.27, *p* < 0.005; Additional file 1: Fig. S5A) and phylogenetic diversity (Faith’s PD, *r* = −0.30, *p* < 0.002; Additional file 1: Fig. S5B). Relative bacterial abundance on leaves (Additional file 1: Fig. S5C) and in larvae did not correlate with larval weight (Additional file 1: Fig. S5D).

### SynCom20 is associated with stronger JA accumulation during larval feeding

We investigated how phyllosphere bacteria and herbivory influence anti-herbivore defence signalling in *A. thaliana*, by quantifying changes in the stress-related phytohormones JA, JA-Ile, SA, and ABA. Plants were inoculated either with PBS (mock-treated control) or with SynCom20 and subsequently exposed to *P. brassicae* larvae. Phytohormone levels were measured at the onset of larval feeding (T0) and 24 h later (T1) in two independent experiments (Additional file 1: Fig. S6).

**Fig. 3:**
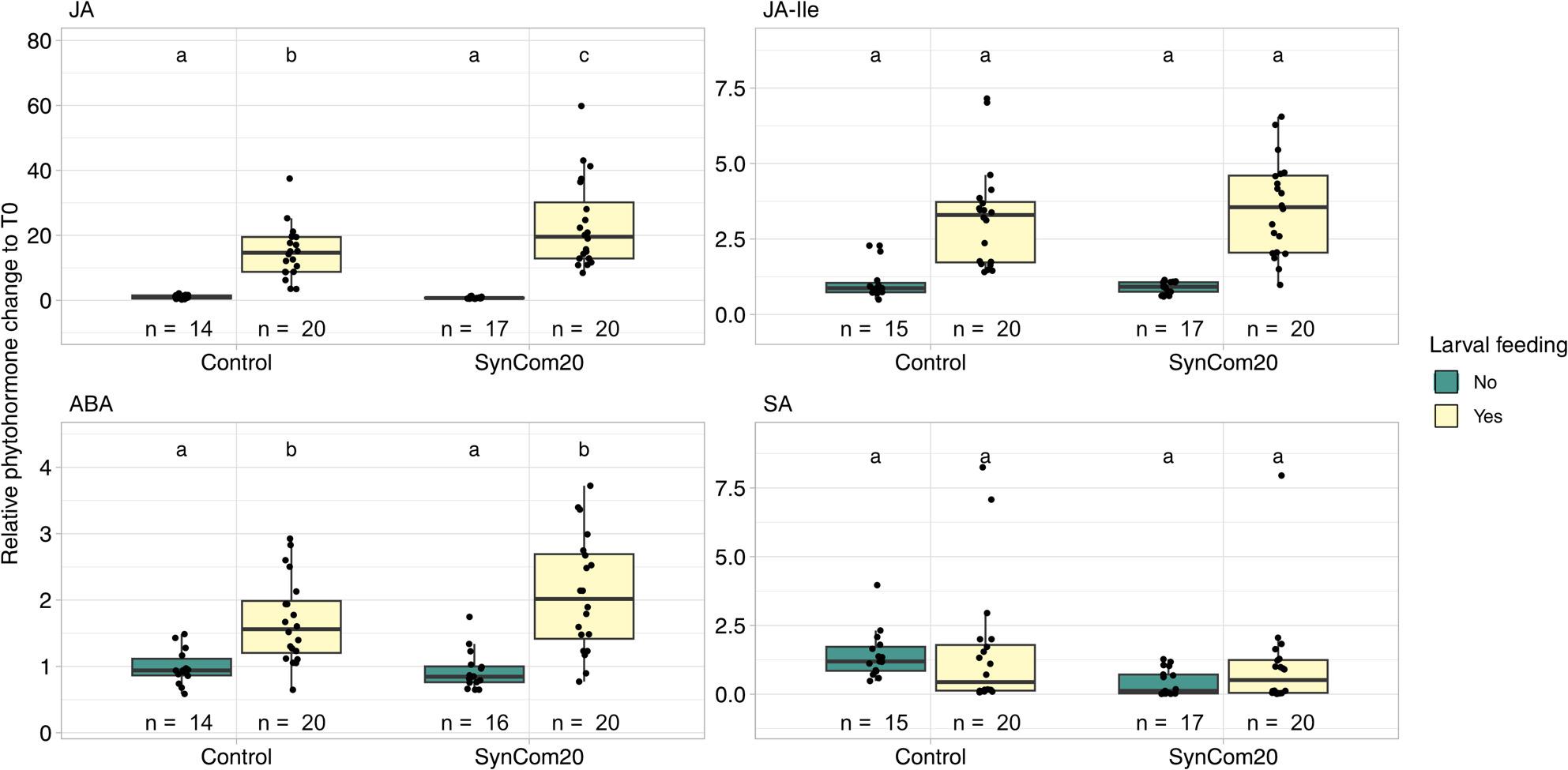
Phytohormone level changes in *A. thaliana* leaves after 24 h of *P. brassicae* larval feeding. Plants were either mock-treated (control) or inoculated with SynCom20, and subsequently either exposed to *Pieris brassicae* larval feeding or not. Phytohormones jasmonic acid (JA), jasmonoyl-isoleucine (JA-Ile), abscisic acid (ABA), and salicylic acid (SA) were quantified at T0 and after 24 h of feeding (T1). Relative concentration changes after 24 h of feeding are shown. Treatments included mock-treated control + no feeding, mock-treated control + feeding, SynCom20 + no feeding, and SynCom20 + feeding. Differences in the number of replicates across treatments reflect sample losses during the phytohormone extraction process and quality filtering. Different letters indicate significant differences among treatments within each phytohormone panel (*p*_adj_ < 0.05) based on generalised linear mixed-effects models (GLMMs) followed by Benjamini–Hochberg-adjusted pairwise comparisons of estimated marginal means

Feeding by *P. brassicae* induced changes in the phytohormone profiles of *A. thaliana* leaves (Fig. 3). After 24 h, JA levels increased in response to herbivory in both mock-treated and SynCom20-inoculated plants, with SynCom20-inoculated plants showing significantly higher JA accumulation under feeding conditions than mock-treated control plants (*p*_adj_ < 0.0001). This pattern was consistent with the raw JA levels at 24 h (Additional file 1: Fig. S6, *p*_adj_ < 0.0001). JA-Ile and ABA levels also increased significantly in response to herbivory but were independent of SynCom20 treatment. SA levels did not differ significantly among treatments. Parallel to the relative changes in phytohormone levels shown in Fig. 3, SynCom20 alone did not affect the raw phytohormone levels at T0 or T1 (Additional file 1: Fig. S6, *p*_adj_ > 0.05). Overall, SynCom20-specific differences were detected only for JA and only under feeding conditions.

### Larval feeding increases bacterial abundance on leaves and alters phyllosphere community composition

**Fig. 4:**
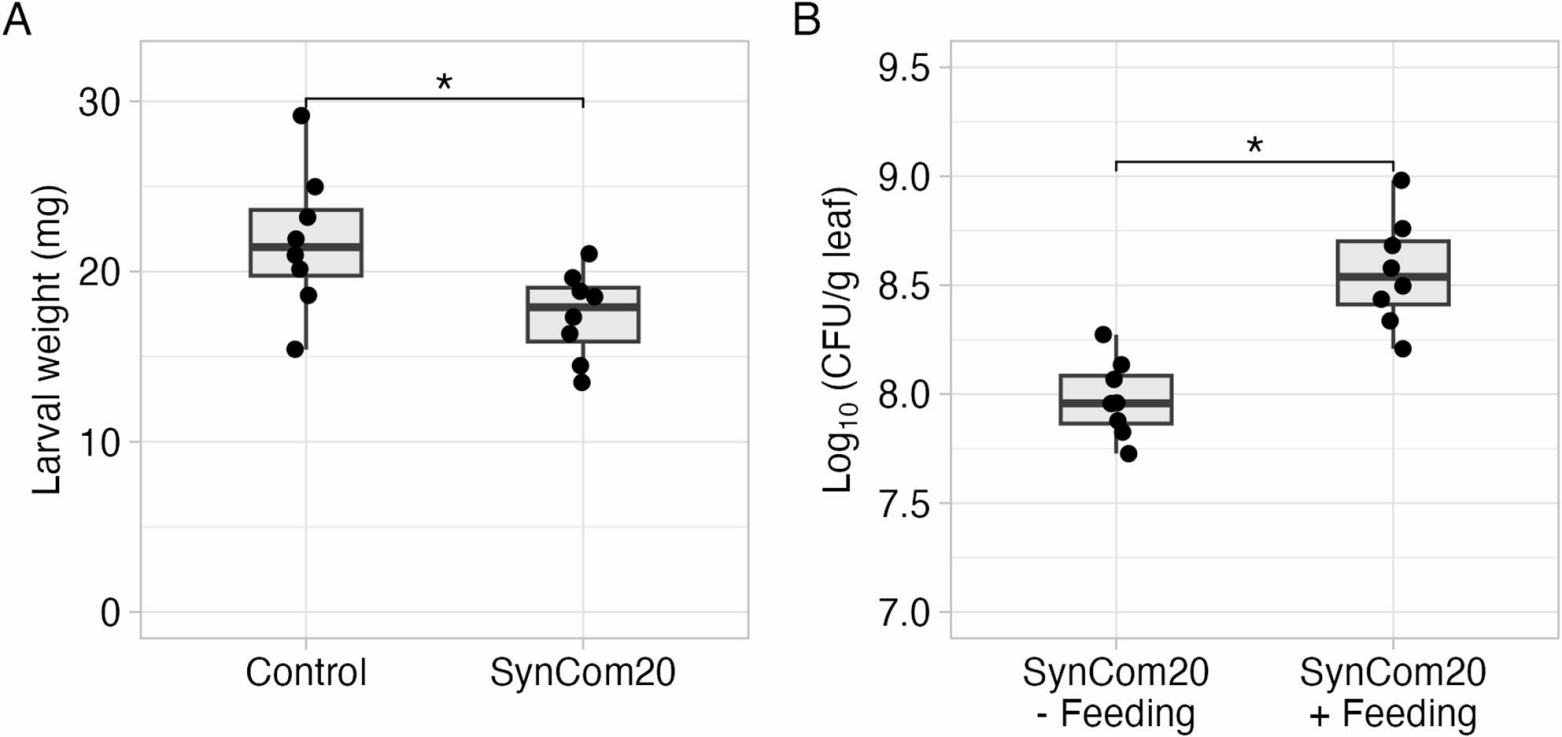
Effect of SynCom20 and herbivory on larval performance and bacterial colonisation. **A** Larval weight of *Pieris brassicae* after feeding for seven days on mock-treated control (*n* = 8) or on SynCom20-inoculated *Arabidopsis thaliana* plants (*n* = 8). **B** Total bacterial abundance on leaves (CFU/g) of SynCom20-inoculated plants exposed or not exposed to *P. brassicae* larvae (*n* = 8, per treatment). Significant differences between groups are indicated by stars (*, α = 0.05; Student’s *t*-test)

To assess how larval feeding influences phyllosphere bacterial abundance, we compared SynCom20-inoculated plants that were fed upon for seven days to plants that did not experience feeding. In line with the results described above (Fig. 2D), larvae feeding on SynCom20-inoculated plants had lower weight than larvae feeding on mock-treated control plants (Fig. 4A; *t*-test, *t*(14) = 2.51, *p* < 0.05). Leaf bacterial loads were approximately fourfold higher on fed compared to non-fed plants, with mean abundances of 4.2 × 10^8^ and 1 × 10^8^ CFU per g of leaf, respectively (Fig. 4B; *t*-test, *t*(14) = 5.44, *p* < 0.05). These results indicate that larval feeding is associated with increased bacterial loads on the leaf surface.

To examine how *P. brassicae* feeding affects SynCom20 community composition on the leaf surface and bacterial acquisition by the larval gut, we performed 16S rRNA gene amplicon sequencing of the V3–V4 region using samples from the same SynCom20 herbivory experiment described above. Axenically grown *A. thaliana* plants were inoculated with SynCom20, and eight days later a subset of boxes received *P. brassicae* neonates. After seven days of feeding, we collected three types of samples: (i) SynCom20-inoculated leaves that had been fed upon, (ii) larvae that fed on those leaves, and (iii) SynCom20-inoculated leaves that were not exposed to larvae. In addition, the original SynCom20 inoculum was sequenced to assess compositional shifts after plant colonisation and herbivory.

**Fig. 5:**
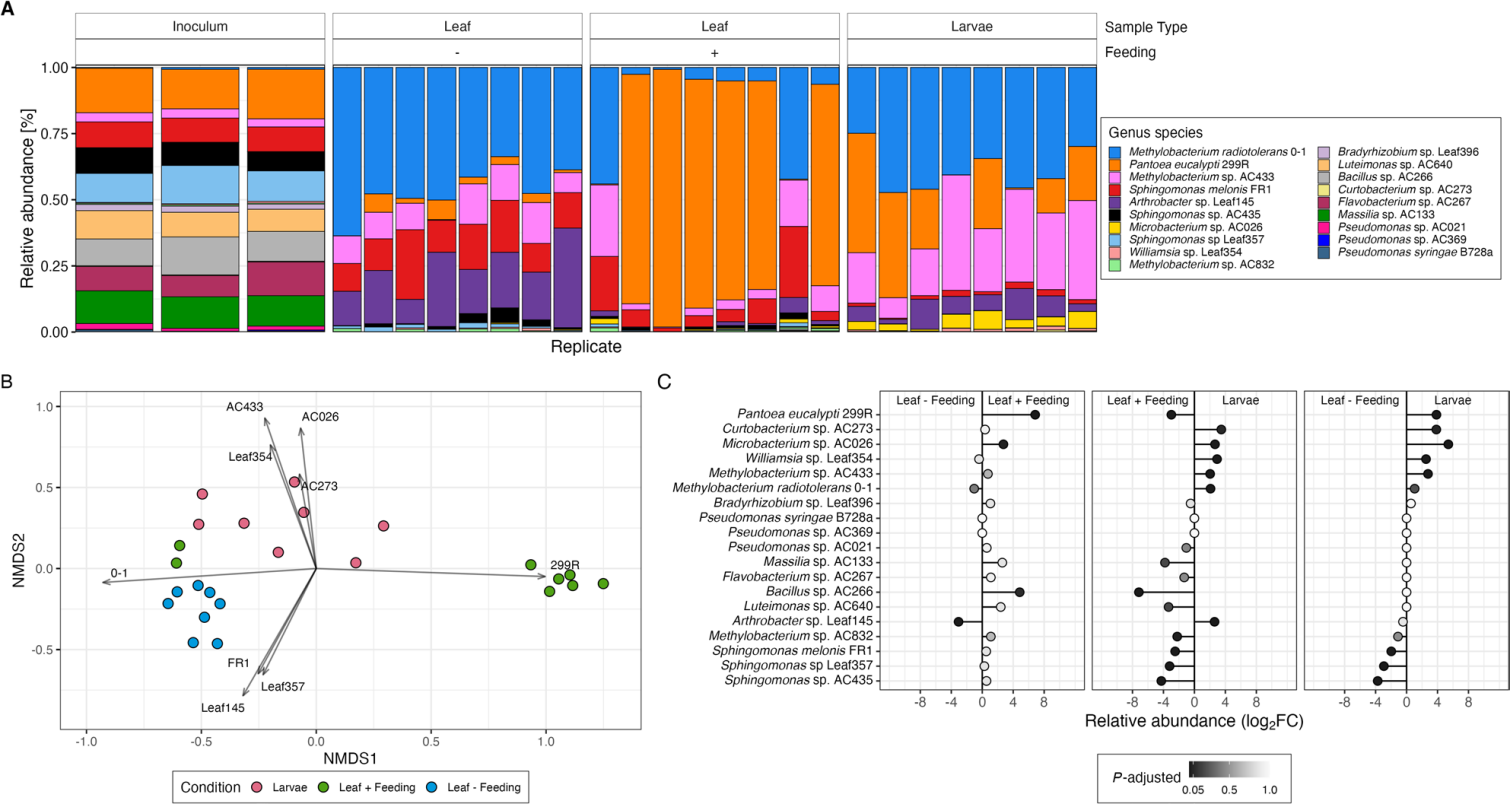
Relative abundance and community composition of SynCom20 under herbivory. Axenically grown *Arabidopsis thaliana* plants were inoculated with SynCom20, and after eight days *Pieris brassicae* neonates were introduced to a subset of boxes and allowed to feed for seven days before sampling (15 days after bacterial inoculation). **A** Relative abundance of bacterial strains across treatments: Inoculum (SynCom20); inoculated non-fed leaves; inoculated fed leaves; larvae after feeding on inoculated plants. **B** NMDS plot on Bray–Curtis distances across treatments. Arrows indicate individual strains driving compositional differences (envfit, *p* < 0.05), with arrow direction positively correlated with abundance. **C** Differential abundance analysis of bacterial strains between treatments, shown as log_2_ fold change (log_2_FC). Left: Herbivory effect on the phyllosphere bacterial community (non-fed leaves vs. fed leaves); centre: Herbivory-altered phyllosphere vs. gut bacterial community (fed leaves vs. larva); right: Overall gut filtering (non-fed leaves vs. larvae). Negative and positive log_2_FC values indicate enrichment in the sample type shown on the left and right of each comparison, respectively

Sample type explained 73.9% of the variance between communities (PERMANOVA, *p* < 0.001). Relative to the original SynCom20 inoculum, both leaf and larval samples showed marked compositional shifts, with several strains becoming undetectable or persisting only at very low abundance (Fig. 5A). Despite a strong overlap in taxa between leaves and larvae, differences in strain abundances resulted in distinct community compositions (Fig. 5A, B).

Overall, differences in community composition between fed and non-fed leaves were driven by a few strains showing strong shifts in relative abundance. For most strains, larval samples showed relative abundances similar to those observed on non-fed leaves. However, some strains were consistently over- or underrepresented in larval samples compared with both fed and non-fed leaves (Fig. 5C).

Larval feeding caused leaf communities to become dominated by *Pantoea eucalypti* 299R, except for two samples that clustered more closely with non-fed leaves or larval communities (Fig. 5A, B). Given the strong increase in total bacterial load on fed leaves, *P. eucalypti* 299R was likely responsible for this proliferation (Figs. 4B and 5A, C). To a lesser extent, *Microbacterium* sp. AC026 and *Bacillus* sp. AC266 also increased in relative abundance in fed compared to non-fed leaves, whereas communities of non-fed leaves were characterised by uniquely high abundances of *Arthrobacter* Leaf145 (Fig. 5C).

By contrast, larval samples were consistently enriched in two *Methylobacterium* strains (*Methylobacterium radiotolerans* 0–1 and *Methylobacterium* AC433), as well as *Microbacterium* sp. AC026, *Curtobacterium* sp. AC273, and *Williamsia* sp. Leaf354, compared with both fed and non-fed leaves (Fig. 5B, C). *Sphingomonas* strains, which were present at low abundance in both fed and non-fed leaves, were strongly underrepresented in larval samples (Fig. 5C).

**Fig. 6:**
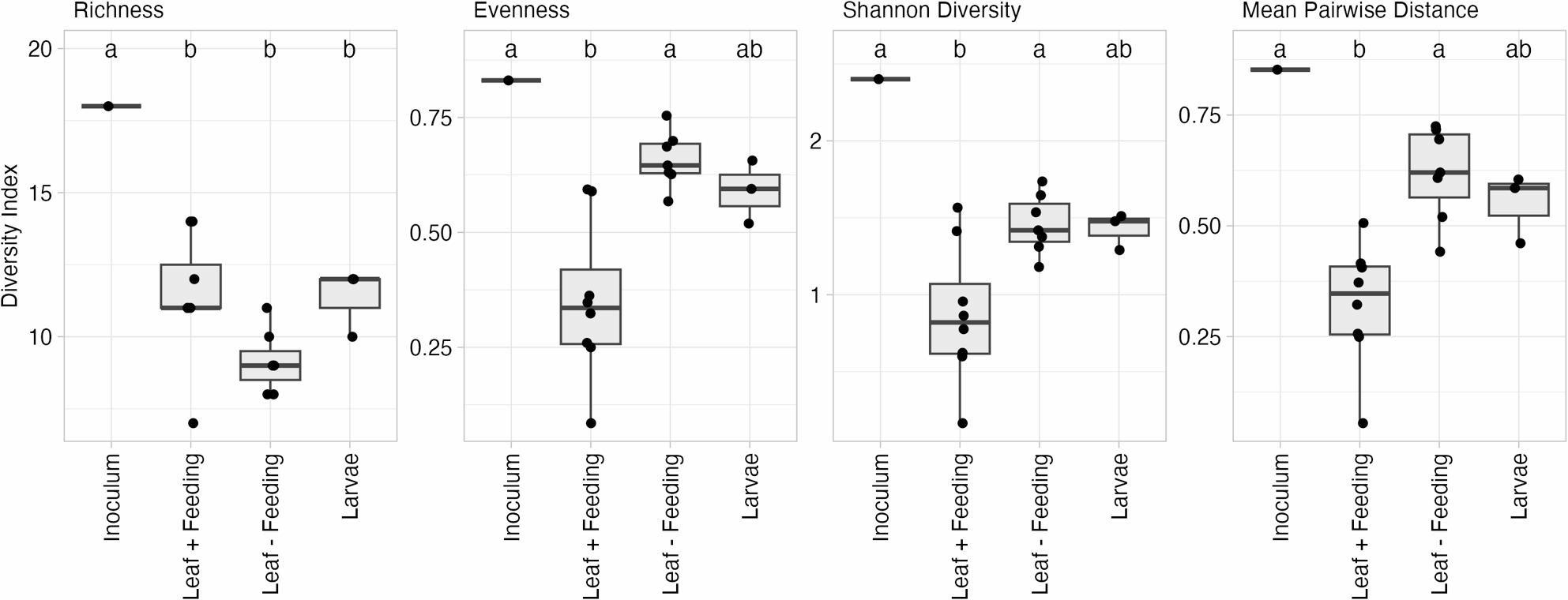
Community diversity metrics of SynCom20 in response to herbivory. *Arabidopsis thaliana* plants were inoculated with SynCom20 and exposed or not exposed to *Pieris brassicae* feeding. Alpha diversity metrics—richness, Shannon’s evenness, Shannon’s diversity, and mean pairwise phylogenetic distance (MPD)—were compared across treatments: Inoculum (SynCom20); inoculated non-fed leaves; inoculated fed leaves; larvae after feeding on inoculated plants. MPD values were normalised to that of an even SynCom20 community where all strains had equal abundance. Different letters indicate significant differences among treatments based on ANOVA followed by Tukey’s *post hoc* test for multiple comparisons

Alpha diversity differed markedly among the bacterial communities from the inoculum, leaves, and larvae (Fig. 6). Richness was highest in the SynCom20 inoculum and decreased in both leaf and larval samples. Evenness was reduced in fed leaf communities compared to non-fed leaves (*p*_adj_ = 0.0256), reflecting the dominance of *P. eucalypti* 299R during herbivory (Fig. 5). Shannon diversity followed a similar pattern, that is, an overall reduction in alpha diversity in fed leaf communities. The mean pairwise phylogenetic distance (MPD), describing the phylogenetic relatedness of strains within a community, was also highest in the inoculum and declined in plant- and larval-associated bacterial communities. Together, these results show that herbivory reshapes SynCom20 communities by increasing overall abundance but reducing evenness and phylogenetic breadth, leading to dominance of specific strains within the leaf microbiota.

## Discussion

Using a functionally gnotobiotic insect–plant system, we showed that resident phyllosphere bacteria and herbivory shape one another, with consequences for defence-associated phytohormone responses, microbial community structure, and insect performance. Increasing bacterial richness in the phyllosphere was associated with reduced *P. brassicae* larval growth, with a significant effect only for the richest community tested (SynCom20), and larval feeding on SynCom20-inoculated plants coincided with stronger JA accumulation than feeding on mock-treated controls. In the other direction, herbivory reshaped the phyllosphere microbiota, increasing bacterial loads and enriching *P. eucalypti* 299R, while larvae acquired only a filtered subset of leaf-associated bacteria, dominated by *Methylobacterium*, *Microbacterium*, *Williamsia*, and *Curtobacterium*. Together, these findings suggest a reciprocal link between phyllosphere bacteria and herbivory: leaf-associated bacteria can modulate plant defence responses and herbivore performance—much as rhizosphere communities do [65–67]—while herbivory reshapes bacterial community structure.

In our system, *P. brassicae* larval growth tended to decrease with increasing bacterial richness in the phyllosphere, with a significant reduction observed only for the richest community (SynCom20) compared to mock-treated control plants. This pattern suggests that higher bacterial richness may enhance the community’s overall impact on plant–insect interactions, potentially through complementary or synergistic effects among strains influencing plant responses [68, 69]. In microbial systems, richer communities often exhibit metabolic complementarity, functional redundancy, and emergent properties that improve community performance and stability [68, 69]. Such emergent properties could enhance the ability of phyllosphere communities to modulate host physiology, thereby altering plant–herbivore interactions. However, it remains unclear whether the observed patterns in our system were driven by overall richness, emergent interactions among community members, or the activity of key taxa within SynCom20. Because plant responses were characterised only for SynCom20 and mock-treated controls, we cannot determine whether these effects reflect bacterial richness per se, the composition of SynCom20, or the activity of specific strains within this community. Candidate strains contributing to the observed effects include those that became most abundant during larval feeding, such as *P. eucalypti* 299R, which is known to be a strong and metabolically versatile competitor in the phyllosphere [70]. These traits may promote its competitive success and capacity to shape community structure during herbivory, potentially affecting plant responses either directly through microbe–host signalling or indirectly by altering the abundance of taxa that influence defence-associated responses. Future work employing broader richness gradients, alternative community compositions, and targeted removal or enrichment of specific members could help disentangle these contributions. Overall, these findings show that phyllosphere bacterial communities composed of strains nonpathogenic to *A. thaliana* can affect herbivore growth, revealing an underappreciated ecological role of resident leaf microbiota in plant anti-herbivore responses.

The reduction in larval weight observed on SynCom20-inoculated plants may be linked to altered defence-associated phytohormone responses. SynCom20-inoculated plants showed stronger JA accumulation during *P. brassicae* feeding than mock-treated control plants. By contrast, JA-Ile and ABA increased in response to herbivory but were not significantly affected by bacterial treatment, while SA remained unchanged. In the absence of herbivory, SynCom20 did not affect phytohormone accumulation. This pattern suggests that SynCom20 altered the plant response to herbivory in a JA-associated manner, without broadly activating SA-associated pathogen-type responses. These results are compatible with a priming-like scenario in which prior exposure to microorganisms enhances the plant’s capacity to respond more rapidly or intensely to a later challenge, such as insect herbivory, compared with unprimed plants [71–73]. Rhizosphere-associated bacteria are well known to induce JA-dependent priming; for example, colonisation of *A. thaliana* roots by *Pseudomonas simiae* WCS417r primes JA/ethylene-regulated defences and enhances resistance against the chewing larvae of *Mamestra brassicae* [74], while plant-growth-promoting rhizobacteria can increase JA accumulation and JA-responsive gene expression in cotton, reducing damage and growth of *Spodoptera exigua* larvae [75, 76]. A similar mechanism may occur in the phyllosphere, where resident nonpathogenic bacteria could sensitise inducible defence-associated responses during herbivory without directly activating strong defences in the absence of feeding. Consistent with this possibility, colonisation of Arabidopsis leaves by the commensal *Sphingomonas melonis* Fr1, a member of SynCom20, has been shown to induce defence-associated transcriptional responses, including the JA/ethylene-responsive marker PDF1.2 [77].

Priming need not be the only route to this response: a second, non-mutually exclusive mechanism may act through changes in bacterial density and localisation during feeding. Feeding by *P. brassicae* increased bacterial loads on the leaf surface, suggesting that higher microbial densities during herbivory could contribute to stronger JA accumulation in SynCom20-inoculated plants. This interpretation is consistent with recent findings showing that plant responses to nonpathogenic phyllosphere bacteria are density-dependent, with high bacterial titres eliciting stronger immune-associated transcriptional responses than low titres [78]. Thus, herbivory-driven proliferation of some SynCom members may increase bacterial titres beyond a level sufficient to enhance plant perception, contributing to a density-dependent defence-alert state. Chewing damage can facilitate bacterial proliferation on leaves [17] and may also provide entry points into internal tissues through wounds [79]. Once bacteria or bacterial molecules reach the apoplast, they can be perceived by pattern-recognition receptors at the plasma membrane, making this compartment an important site for defence activation [80, 81]. Therefore, stronger JA accumulation in SynCom20-inoculated plants during feeding could result from the combined effects of herbivory-induced bacterial proliferation and increased bacterial access to wounded plant tissues. Such processes may explain how nonpathogenic phyllosphere bacteria, while eliciting no phytohormone accumulation under non-feeding conditions, modulate inducible plant responses during herbivory.

Although the co-occurrence of reduced larval growth and stronger JA accumulation on SynCom20-inoculated plants points towards a SynCom-associated change in the plant–insect interaction, direct effects of ingested bacteria on larvae cannot be excluded. In our system, bacterial abundance on the leaf surface was not correlated with larval weight, and no visible disease symptoms were observed in larvae. However, these observations do not rule out sublethal bacterial effects on larval physiology, digestion, gut function, feeding behaviour, or food conversion efficiency. Bacteria may also have altered leaf acceptability by changing leaf surface cues, microbial metabolites, or plant tissue quality. Such effects are not necessarily independent of plant defence-associated responses, because defence-related changes in tissue quality could themselves influence larval intake and growth [12, 82]. The hormonal results should also be interpreted cautiously: while SynCom20-inoculated plants showed stronger JA accumulation during *P. brassicae* feeding, JA-Ile, the bioactive jasmonate most directly linked to anti-herbivore defence, did not show a clear SynCom20-specific increase, and SA levels remained unchanged across treatments. Thus, although the specific herbivory-associated cues involved remain unresolved, the comparison between mock-treated and SynCom20-inoculated plants under the same feeding treatment indicates that SynCom20 modified the plant phytohormone response during herbivory. This altered response provides a plausible link between phyllosphere colonisation and reduced larval growth, but the underlying mechanism remains unresolved.

Beyond these effects of bacteria on the herbivore, our data also reveal the reverse: herbivory reshaped bacterial community assembly on leaves. Following SynCom20 inoculation and a subsequent feeding period, *P. brassicae* herbivory led to communities distinct from those observed on non-fed plants. This shift was characterised by increased bacterial loads, reduced evenness, and dominance of a subset of strains, suggesting that feeding created conditions favouring particular community members. Wounding during feeding likely modifies the physicochemical environment of the leaf [17], potentially through the release of intracellular metabolites such as soluble sugars from damaged cells. Insect frass, which can contain labile carbon and nitrogen [83], may further contribute nutrient-rich material to the leaf surface, together creating conditions that favour the proliferation of metabolically versatile taxa. In addition to these direct effects of wounding and nutrient availability, herbivory-induced changes in defence-associated phytohormone responses may also contribute to community restructuring [17]. Overall, these results show that herbivory alters which bacterial taxa persist and proliferate on leaves, thereby changing the bacterial community encountered by feeding larvae. Future experiments tracking individual SynCom strains across leaves, larvae, and frass, combined with controlled wounding and frass application, would help distinguish the contributions of wound-released nutrients, frass deposition, and larva-mediated bacterial return to the leaf surface.

The herbivory-associated shift in leaf community composition was driven mainly by changes in the relative abundance of a few strains. Fed leaves became dominated by *P. eucalypti 299R*, consistent with the strong increase in total bacterial abundance observed after larval feeding. To a lesser extent, *Microbacterium* sp. AC026 and *Bacillus* sp. AC266 also increased in relative abundance on fed leaves, whereas non-fed leaves were characterised by higher relative abundance of *Arthrobacter* sp. Leaf145. These patterns suggest that herbivory did not uniformly promote the entire SynCom20 community, but instead favoured specific taxa able to exploit the altered leaf environment. Communities on leaves and in larvae also became less diverse and more phylogenetically clustered than the original inoculum, indicating strong environmental filtering after inoculation and during herbivory. Together, these shifts show that herbivore feeding changes not only total bacterial abundance, but also the identity and relative dominance of phyllosphere bacteria available for ingestion by larvae.

Comparison of bacterial assemblages among the SynCom20 inoculum, leaf surfaces, and larval guts further revealed strong compositional shifts as bacteria transitioned from the inoculum to the phyllosphere and subsequently to the larval gut. Only a subset of strains persisted on leaves or in larvae, consistent with strong host- and environment-mediated filtering [23, 84]. Larval communities shared several taxa with leaf communities but remained compositionally distinct, indicating that ingestion and passage through the gut further reshaped community structure. Larvae were enriched in *Methylobacterium radiotolerans* 0–1, *Methylobacterium* sp. AC433, *Microbacterium* sp. AC026, *Curtobacterium* sp. AC273, and *Williamsia* sp. Leaf354, whereas *Sphingomonas* strains were strongly underrepresented. These patterns suggest that the larval gut does not passively reflect the bacterial community present on leaves, but instead selects for a distinct subset of phyllosphere-derived bacteria.

The biological consequences of this gut-associated bacterial subset remain unclear. Lepidopteran larvae generally lack stable resident gut microbiota, likely because the gut is highly alkaline, has rapid food transit, and provides limited opportunities for persistent colonisation [23, 84, 85]. Nevertheless, the enrichment of specific bacterial taxa in *P. brassicae* larvae suggests that some phyllosphere-derived bacteria may persist transiently or proliferate during gut passage rather than merely passing through with ingested leaf material. These bacteria could interact with larval physiology or metabolism, for example by modifying ingested plant material, producing metabolites, or competing with other microorganisms, although these possibilities were not tested here. Thus, the larval gut appears to act as a selective environment for a subset of leaf-associated bacteria, but whether these bacteria affect larval performance, microbial interactions, or susceptibility to other microorganisms remains to be determined.

Reintroduction of gut-associated bacteria onto the leaf surface via frass deposition or regurgitation may also contribute to phyllosphere community dynamics, but our results suggest that this effect was smaller than the direct impact of herbivory itself. Bacterial communities on non-fed leaves were more similar to those found in larvae than to those on fed leaves, indicating that larva-associated bacteria did not substantially override the herbivory-driven shift in leaf community structure. By contrast, feeding was associated with major changes in bacterial abundance and composition, likely driven by tissue damage, nutrient leakage, and changes in leaf physiology and defence-associated responses [17]. These results suggest that herbivory reshapes the phyllosphere primarily by modifying habitat conditions and resource availability, rather than simply by returning gut-associated bacteria to the leaf surface. Overall, the feedback between herbivory and the phyllosphere microbiota appears asymmetric in character: leaf-associated bacteria can influence plant responses and herbivore performance indirectly, whereas herbivory acts more directly on microbial community structure, reshaping bacterial abundance and composition on the leaf.

## Conclusions

This study reveals that nonpathogenic phyllosphere bacteria can influence defence-associated phytohormone responses and herbivore performance, demonstrating an overlooked ecological role of resident leaf microbiota in plant–insect interactions. Increasing bacterial richness was associated with reduced larval growth, while SynCom20 was associated with stronger JA accumulation during feeding. Because phytohormones were measured only for SynCom20, future work will be needed to determine whether this response reflects community richness, community composition, or the activity of specific strains. Herbivory, in turn, reshaped the phyllosphere community and increased bacterial abundance, pointing to reciprocal feedback in which microbial activity and feeding jointly shape plant physiology and microbiome structure. Unravelling the molecular and ecological mechanisms underlying these feedbacks, particularly how bacterial localisation, density, and perception contribute to defence-associated responses, will be key to understanding how aboveground microbiomes shape plant responses to insect attack.

Finally, the functionally gnotobiotic insect–plant system developed here provides a reproducible and scalable framework for testing defined phyllosphere communities under low-background microbial conditions. Its controlled design enables the systematic disentanglement of mechanisms linking microbial community structure, plant defence-associated responses, and herbivore performance, paving the way for mechanistic and translational studies on the ecological functions of aboveground microbiomes.

## Supplementary Information

Below is the link to the electronic supplementary material.

**Additional file 1**.

## Data Availability

The datasets and materials supporting the conclusions of this article are available in thefollowing repositories: The 16S amplicon sequencing data are in the European Nucleotide Archive (ENA) repository, accession number PRJEB104625(https://www.ebi.ac.uk/ena/browser/view/PRJEB104625). The additional datasets are available in Zenodo (10.5281/zenodo.17822324). TheR scripts used for data analysis are accessible in the GitHub repository (https://github.com/relab-fuberlin/mueller_arabidopsis_pieris_syncom).
