# Supplementary material for: From the leaf to the gut and back again: using synthetic bacterial communities to trace the fate and influence of phyllosphere bacteria in an Arabidopsis–Pieris brassicae system
Source: Environ Microbiome. 2026 Aug 6;21:96. doi: 10.1186/s40793-026-00947-y (PMC13445934; doi:10.1186/s40793-026-00947-y)
Supplement: Supplementary file 1 [file 40793_2026_947_MOESM1_ESM.docx]

**Additional File 1. Supplementary Figures and Tables**

This file contains Supplementary Figures S1–S6 and Supplementary Table S1 referenced in the manuscript:

**From the leaf to the gut and back again: using synthetic bacterial communities to trace the fate and influence of phyllosphere bacteria in an Arabidopsis – *Pieris brassicae* system**

Moritz Müller, Maryse Huve, Michael Kunzler, Mitja N. P. Remus-Emsermann, Rudolf O. Schlechter, Luis R. Paniagua Voirol

**Contents**

- **Supplementary Figure S1. Composition of SynComs used in each experimental run.**
- **Supplementary Figure S2. Classification of 16S rRNA gene sequencing reads.**
- **Supplementary Figure S3. Pairwise V3-V4 16S rRNA sequence identity among SynCom20 strains.**
- **Supplementary Figure S4. Effect of Magenta Box vs. Pot growth system in Pieris brassicae larval survival and biomass.**
- **Supplementary Figure S5. Correlations between normalised larval weight, SynCom properties, and bacterial abundance.**
- **Supplementary Figure S6. Phytohormone levels in *A. thaliana* leaves across independent experiments.**
- **Supplementary Table S1. Bacterial strain pool for SynCom assembly.**

**Supplementary Figures**

**Supplementary Figure S1.**

**Figure S1. Composition of SynComs used in each experimental run.** For all independent experiments in which SynCom5 (5 strains), SynCom10 (10 strains) and SynCom20 (20 strains) were used, the specific strains present in the SynCom5 and SynCom10 bacterial communities were drawn randomly out of the SynCom20 strain pool. Strains present in a SynCom used in an independent experiment are coloured dark. The number of biological replicates (N) per treatment (SynCom5, SynCom10, and SynCom20) is indicated for each independent experiment. The number of replicates in the mock controls matched the N of the corresponding experiment. Experiment 1 did not include SynCom10.

**Supplementary Figure S2.**

**Figure S2. Classification of 16S rRNA gene sequencing reads.** OTUs were classified using the SILVA rRNA database grouped by sample type. Kitome samples are reagent-only extraction negative controls processed alongside the biological samples to monitor bacterial DNA originating from the extraction reagents. Chloroplast reads are shown in green, mitochondrial reads in orange, SynCom20 reads in teal, and other/unclassified reads in grey. (A) Total number of 16S rRNA gene amplicon reads. (B) Relative abundance of 16S rRNA gene amplicon reads. (C) Relative abundance of 16S rRNA gene amplicon reads after removal of chloroplast and mitochondrial reads.

**Supplementary Figure S3.**

**Figure S3. Pairwise V3-V4 16S rRNA sequence identity among SynCom20 strains.** Heatmap showing percent pairwise sequence identity between all 20 strains. Several strain pairs share identity above 98%, indicating a lower discriminatory power of the V3-V4 region for resolving these strains by amplicon sequencing.

**Supplementary Figure S4.**

**Figure S4: Effect of the Magenta Box vs. Pot growth system in *Pieris brassicae* larval survival and biomass.** (A) Larval survival, Fisher’s exact test (n = 7); (B) Mean larval weight (n = 7). Black points represent individual biological replicates, red points represent treatment means, and red error bars indicate confidence intervals (α = 0.05). Significant differences between groups are indicated by an asterisk (*p* < 0.05; Wilcoxon signed-rank test).

**Supplementary Figure S5.**

**Figure S5. Correlations between normalised larval weight, SynCom properties, and bacterial abundance.** In all panels, Spearman’s correlations (R) were calculated using larval weight normalised to the mean weight of larvae in the corresponding mock-treated control group within each independent experiment. Each point represents one biological replicate. Colours indicate the bacterial treatment: mock-treated control (red), SynCom5 (blue), SynCom10 (green), and SynCom20 (purple). Black lines show fitted linear trends for visualisation. (A) SynCom richness, expressed as the number of strains per SynCom. (B) SynCom phylogenetic diversity, expressed as Faith’s phylogenetic diversity (PD). (C) Bacterial abundance on leaves after larval feeding, expressed as log10 CFU per gram of leaf fresh weight. (D) Bacterial abundance in larvae, expressed as log10 CFU per gram of larval fresh weight.

**Supplementary Figure S6.**

**Figure S6. Phytohormone levels in *A. thaliana* leaves across independent experiments.** Raw phytohormone concentrations (ng/g leaf fresh weight) of jasmonic acid (JA), jasmonoyl-isoleucine (JA-Ile), abscisic acid (ABA), and salicylic acid (SA) were measured at baseline (T0, eight days after bacterial inoculation and immediately before larval introduction) and 24 h later (T1). Plants were either mock-treated with PBS (Control) or inoculated with SynCom20, and subjected to larval feeding (Yes) or not (No). Data are shown separately for each independent experimental run (1 and 2). Individual data points representing biological replicates are overlaid on boxplots.

**Supplementary Tables**

**Supplementary Table S1.**

**Table S1. Bacterial strain pool used for SynCom assembly.** For each strain, taxonomic classification, genome assembly accession number, OD600 used during SynCom assembly, and growth medium are provided.

| **Phylum** | **Class** | **Order** | **Family** | **Genus** | **Species** | **Strain** | **Accession** | **OD600 at assembly*** | **Growth medium** |
| --- | --- | --- | --- | --- | --- | --- | --- | --- | --- |
| Actinomycetota | Actinomycetia | Micrococcales | Microbacteriaceae | *Curtobacterium* | *sp.* | AC273 | GCA_979253735.1 | 1 | liquid R2A |
| Actinomycetota | Actinomycetia | Micrococcales | Microbacteriaceae | *Microbacterium* | *sp.* | AC026 | GCA_979258475.1 | 0.1 | liquid R2A |
| Actinomycetota | Actinomycetia | Micrococcales | Micrococcaceae | *Arthrobacter* | *sp.* | Leaf145 | GCA_001423565.1 | 0.1 | liquid R2A |
| Actinomycetota | Actinomycetia | Mycobacteriales | Nocardiaceae | *Rhodococcus* | *sp.* | Leaf225 | GCA_001426145.1 | 1 | liquid R2A |
| Actinomycetota | Actinomycetia | Mycobacteriales | Nocardiaceae | *Williamsia* | *sp.* | Leaf354 | GCA_001424365.1 | 1 | R2A agar |
| Bacillota | Bacilli | Bacillales | Bacillaceae | *Bacillus* | *sp.* | AC266 | GCA_979253805.1 | 10 | liquid R2A |
| Bacteroidota | Flavobacteriia | Flavobacteriales | Flavobacteriaceae | *Flavobacterium* | *sp.* | AC267 | GCA_979258715.1 | 0.1 | R2A agar |
| Pseudomonadota | Alphaproteobacteria | Hyphomicrobiales | Methylobacteriaceae | *Methylobacterium* | *sp.* | AC433 | GCA_979253015.1 | 1 | R2A agar |
| Pseudomonadota | Alphaproteobacteria | Hyphomicrobiales | Methylobacteriaceae | *Methylobacterium* | *sp.* | AC832 | GCA_979256025.1 | 1 | R2A agar |
| Pseudomonadota | Alphaproteobacteria | Hyphomicrobiales | Methylobacteriaceae | *Methylobacterium* | *radiotolerans* | 0-1 | GCA_000019725.1 | 1 | liquid R2A |
| Pseudomonadota | Alphaproteobacteria | Hyphomicrobiales | Nitrobacteraceae | *Bradyrhizobium* | *sp.* | Leaf396 | GCA_001424605.1 | 1 | liquid R2A |
| Pseudomonadota | Alphaproteobacteria | Sphingomonadales | Sphingomonadaceae | *Sphingomonas* | *sp.* | AC435 | GCA_979253005.1 | 1 | R2A agar |
| Pseudomonadota | Alphaproteobacteria | Sphingomonadales | Sphingomonadaceae | *Sphingomonas* | *sp.* | Leaf357 | GCA_001423845.1 | 1 | liquid R2A |
| Pseudomonadota | Alphaproteobacteria | Sphingomonadales | Sphingomonadaceae | *Sphingomonas* | *melonis* | FR1 | GCA_000419565.1 | 1 | liquid R2A |
| Pseudomonadota | Betaproteobacteria | Burkholderiales | Oxalobacteraceae | *Massilia* | *sp.* | AC133 | GCA_979253245.1 | 1 | liquid R2A |
| Pseudomonadota | Gammaproteobacteria | Enterobacterales | Erwiniaceae | *Pantoea* | *eucalypti* | 299R | GCA_000330765.1 | 1 | liquid R2A |
| Pseudomonadota | Gammaproteobacteria | Lysobacterales | Lysobacteraceae | *Luteimonas* | *sp.* | AC640 | GCA_979256585.1 | 1 | R2A agar |
| Pseudomonadota | Gammaproteobacteria | Pseudomonadales | Pseudomonadaceae | *Pseudomonas* | *sp.* | AC021 | GCA_979257895.1 | 1 | liquid R2A |
| Pseudomonadota | Gammaproteobacteria | Pseudomonadales | Pseudomonadaceae | *Pseudomonas* | *sp.* | AC369 | GCA_979258735.1 | 1 | liquid R2A |
| Pseudomonadota | Gammaproteobacteria | Pseudomonadales | Pseudomonadaceae | *Pseudomonas* | *syringae* | B728a | GCA_000012245.1 | 1 | liquid R2A |

*During SynCom assembly, equal volumes of the strain suspensions were combined, with strain-specific OD600 values adjusted to account for differences in the relationship between optical density and viable cell concentration.
